# Late Spinal Implant Infection caused by *Cutibacterium acnes*

**DOI:** 10.7150/jbji.36802

**Published:** 2019-07-25

**Authors:** Valentin Gisler, Lorin Benneker, Parham Sendi

**Affiliations:** 1 Department of Infectious Diseases, Bern University Hospital, Bern, Switzerland; 2 Clinic of Infectious Diseases and Hospital Hygiene, Department of Internal Medicine, Kantonsspital Aarau, Aarau, Switzerland. (Current address).; 3 Department for Orthopaedic Surgery, Spine Unit, Inselspital, University Hospital of Bern, Bern, Switzerland; 4 Institute for Infectious Diseases, University of Bern, Bern, Switzerland; 5 Department of Orthopaedics and Traumatology, University Hospital Basel, Basel, Switzerland.; 6 Department of Infectious Diseases and Hospital Epidemiology, University Hospital Basel, University Basel, Basel, Switzerland

## Abstract

*Cutibacterium* spp. have been frequently associated with foreign-body material infections. The vast majority of these infections occur via the exogenous route. Rarely, haematogenous infections occur, possibly seeding from pilosebaceous glands. A late spinal implant-associated infection is presented in this case report, and the possible sources of haematogenous seeding are discussed.

## Introduction

*Cutibacterium* spp. (formerly *Propionibacterium* spp.) is slow-growing, anaerobic Gram-positive rods. In the field of bone and joint infections, they are associated with foreign body material infections, in particular with periprosthetic shoulder joint infection 1. The vast majority of these infectious occur via the exogenous route, namely through inoculation of the microorganisms during surgery or in the early postoperative period. Due to their subtle symptoms, the diagnosis is typically made several months or years after implantation. These biofilm-associated infections are so called “low grade infection” 2. Here, we present a rare case of late spinal implant-associated infection and discuss the possible sources of haematogenous seeding.

## Case Report

In September 2015, a 71-year-old man was referred to our hospital because of steadily increasing lumbar back pain over the past months.

His medical history included total thyroidectomy for papillary cancer in 1986, dynamic stabilization over vertebral segments L2 to S1 for degenerative spine disease in 1996, and a *transurethral resection of* the *prostate for benign prostate hyperplasia* in 2004.

Ten months (November 2014) before referral, the patient consulted an urologist because of urinary obstruction. A urinary tract infection caused by *Escherichia coli* (fully susceptible to all tested antibiotics), a meatus stenosis and an enlarged prostate was diagnosed. Antimicrobial treatment with ciprofloxacin (500 mg bid for 7 days) was initiated, and seven days later, an open meatotomy and a prostate biopsy performed. Prior to the intervention, antimicrobial prophylaxis with cefuroxime (1.5 intravenously) was administered. The postoperative course was uneventful, and the patient discharged two days later. The histopathological analysis of the prostate demonstrated hyperplasia of the glands, stroma and smoot muscles. In addition, small areas of chronic and acute inflammation were seen in the tissue samples. No microbiological examination of the samples was performed. One month later, he claimed of lumbar back pain. The symptom was steadily increasing. Ten months later, he was referred to our institution.

**Figure 1:**
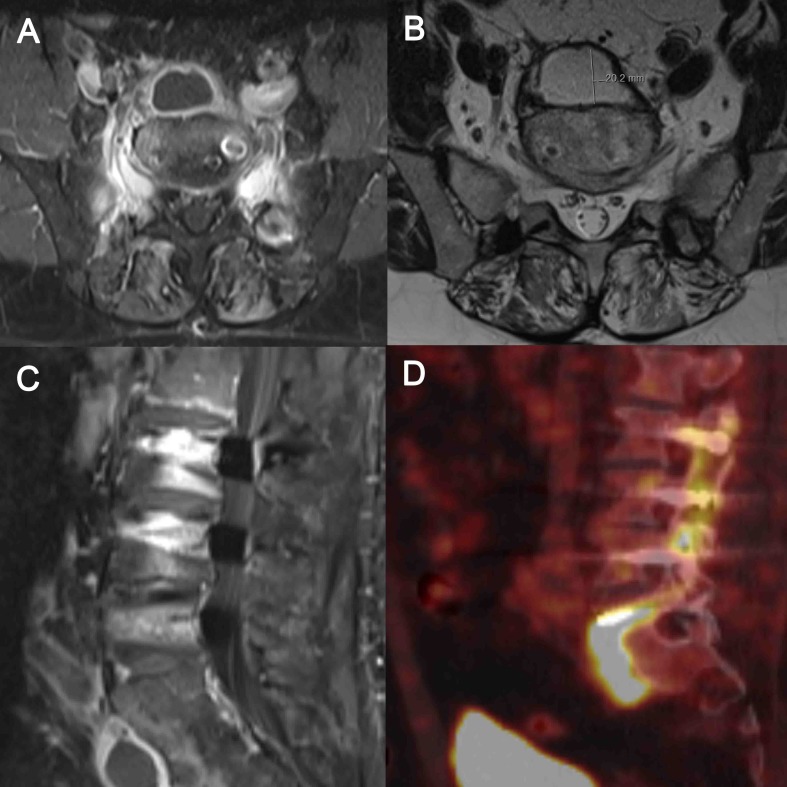
MRI and PET-CT indicated a small abscess anterior (20.2 mm in diameter, B) to segment L5/S1. A, transversal T1 sequence of MRI with contrast medium. B, transversal T2 sequence of MRI with contrast medium. C, sagittal T1 sequence of MRI with contrast medium. D, PET-Scan of the lumbar spine.

On physical examination, the patient's lumbar spine was tender. MRI and PET-CT indicated a small abscess anterior to segment L5/S1 (Figure 1). Intraoperatively, pus was evident. After drainage of the abscess, foreign body material on vertebral levels L2 to L5 were exchanged (1-stage exchange). The metalwork on vertebral level S1 was removed without re-implanting new foreign material. Empiric intravenous antimicrobial agents was administered (vancomycin 15 mg/kg BID plus ceftriaxone 2 g OD). No growth of microorganisms was detected in biopsy samples after 3 days of incubation, but results of histopathological examinations were consistent with infection. A 16S DNA PCR examination on biopsy samples revealed *Cutibacterium acnes.* Sonication of the implants revealed growth of *Cutibacterium acnes* (> 1000 CFU/mL), and the same microorganism grew in 6 of 6 biopsies after 7 days of incubation. The further clinical course was complicated by a transient renal failure, most likely due to accumulation of nephrotoxic drugs. The 3-months course of effective postoperative antimicrobial treatment was completed with oral amoxicillin (1g three times per day). No relapse of infection was noted 36 months after the infection episode.

## Discussion

Haematogenous *Cutibacterium* infections are rarely reported and difficult to prove. However, *Cutibacterium* infection via the haematogenous route is possible, as previously demonstrated in a rabbit model 3. Growth of *Cutibacterium acnes* in blood cultures is frequently interpreted as contaminant. Park et al. 4 reported that in only 3.5% (18/522) of patients with *Cutibacterium* bacteraemia, the microorganism was interpreted as causative pathogen. A third of them (6/18) had undergone invasive procedure prior to development of bacteraemia. These interventions included transarterial embolization (2 cases), radiofrequency ablation, bronchoscopy, cystoscopy and pericardiocentesis. Thus, the translocation from colonizing flora into the blood during or after the intervention was suggested in these cases. Kestler et al. 5, investigated 14 infective endocarditis (IE) cases caused by *Cutibacterium* spp., (13 [93%] male patients). In two of them, a native valve was affected (male patients, aged 84 and 85 years old), suggesting a haematogenous pathogenesis. Banzon et al. 6 presented their series on IE caused by *Cutibacterium* spp. in 2017. The authors reported that all patients were male. The time interval from prosthetic valve or ring placement to the diagnosis of IE ranged from 4 months to 12 years. The pathogenesis in very late infections (i.e.; beyond 3 years after surgery) can be attributed to either reactivation of dormant microorganisms or - more likely - to bacterial seeding from a distant focus. In implant-associated infection, a haematogenous pathogenesis is often postulated when the time interval from surgery to onset of symptoms is beyond two or three years, although these data stem mainly from *S.aureus* 7, 8. Taken together, these arguments underline that haematogenous infection caused by *Cutibacterium* spp. is possible though rare, and there is a male predominance.

The source for haematogenous seeding of *Cutibacterium* spp*.* is less clear. Banzon et al. 6 argue that *Cutibacterium* infections may originate from bacterial seeding at the time of surgery and that the male predominance has been attributed to the higher concentration of pilosebaceous glands in this gender. In our case, the diagnosis was made 19 years after implantation of the foreign-body material. Although very late reactivation of dominant microorganism cannot be excluded, there are arguments for haematogenous seeding. Firstly, symptoms started shortly after the urological intervention and increased gradually until diagnosis. Park et al. 4 reported one case of *Cutibacterium* bacteraemia after cystoscopy. Secondly, the infection site suggests an association with the perineal origin through the Batson plexus. The venous plexus has been described in the pathogenesis of metastatic spread in prostate cancer and of spondylodiscitis caused by Gram-negative bacteria. Thirdly, numerous publications have indicated the association of *Cutibacterium* spp*.* and benign prostate hyperplasia, prostate inflammation or prostate cancer 9-13. Alexeyev et al. 14 studied archival prostate samples from 352 patients with benign prostate hyperplasia with 16S DNA PCR and reported *Cutibacterium spp.* as the most commonly found bacteria (23% of PCR positive samples). Though, the choice of 16S PCR primers 15 and the location of specimen sampling (e.g., samples exposed to the urethra) may decrease the molecular test sensitivity 16. Cavarretta et al. 17 characterized the microbiome in 16 prostatectomy specimens and reported that *Cutibacterium* spp. were the most abundant genus found. Of note, the antibiotic susceptibility in prostate-derived *Cutibacterium* spp. isolates is not different than those obtained at different anatomic sites (e.g.; shoulder) 18.

It is conceivable that manipulation at a prostate that is colonized with *Cutibacterium* spp*.* can lead to either a clinically obvious or a silent bacteraemia. Theoretically, this hypothesis is consistent with the observation of a male predominance in *Cutibacterium*-associated infections. It does not conflict with the higher concentration of pilosebaceous glands in men but adds an additional argument. Because no bacteraemia is documented in our case and the prostate biopsy was not examined microbiologically, we cannot proof our hypothesis of haematogenous seeding. Also, we cannot identify the precise time point of haematogenous seeding, only the onset of symptoms. The preceding treatment with ciprofloxacin for urinary tract infection and the antimicrobial prophylaxis with cefuroxime, however, did not prevent the infection. Nonetheless, the case raises the question of whether or not the prostate is a possible source for haematogenous seeding.

In conclusion, we presented a late spinal implant-associated infection caused by *Cutibacterium acnes*. The time interval from implantation to onset of symptoms and to infection diagnosis (19 years) suggests a haematogenous pathogenesis. The source of postulated bacteraemia is unknown but development of bacteraemia after an invasive procedure is plausible. Pilosebaceous glands are a typical source of *Cutibacterium acnes* colonization. This case has raised the question of whether or not the prostate is another possible source. It also illustrates that diligent work-up is needed, if symptoms arise around an implant after an invasive intervention.
